# Supplementary figures and images for: Presence and stability of SARS-CoV-2 on environmental currency and money cards in Utah reveals a lack of live virus
Source: PLoS One. 2022 Jan 25;17(1):e0263025. doi: 10.1371/journal.pone.0263025 (PMC8789161; doi:10.1371/journal.pone.0263025)

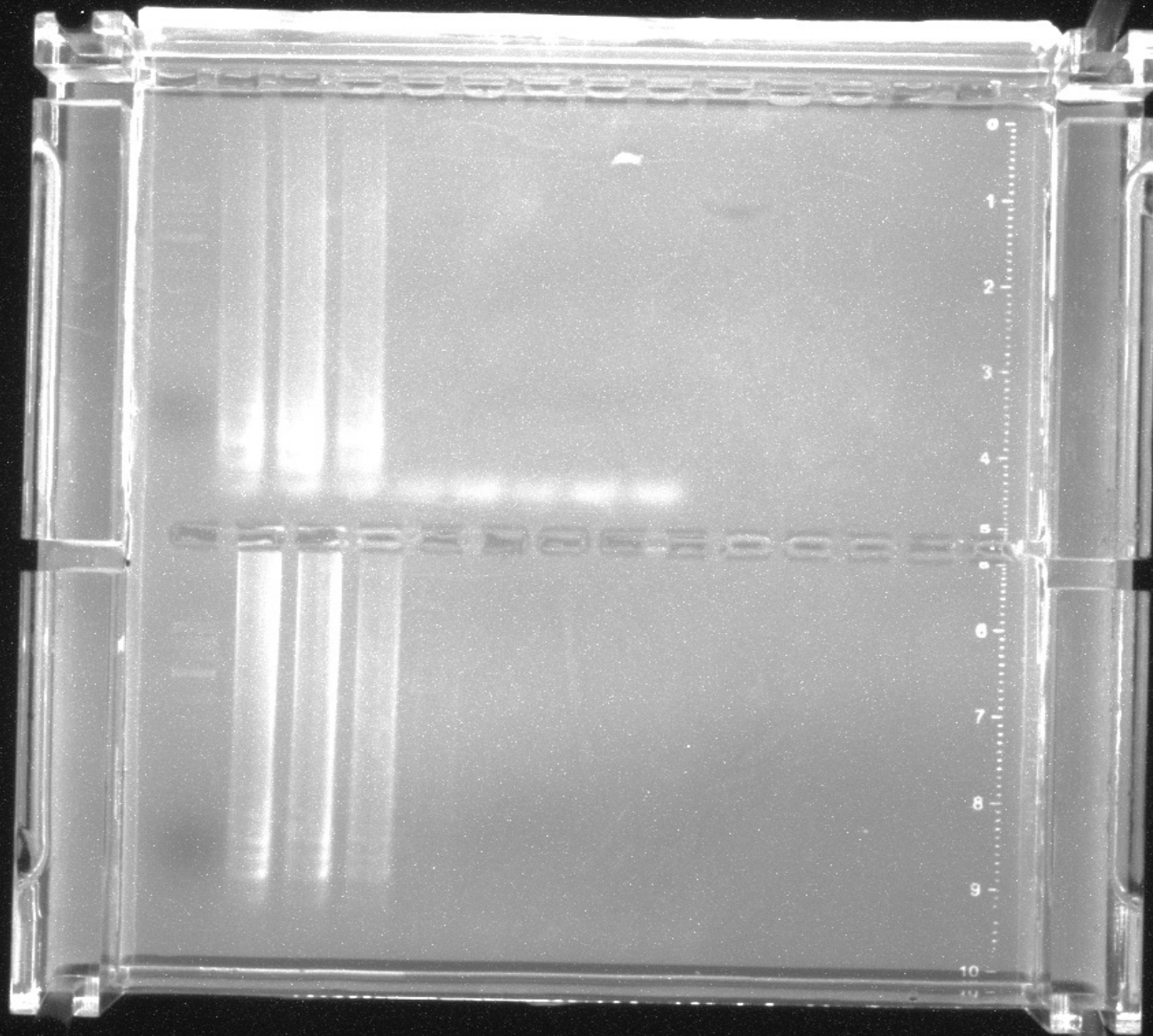

Supplement: S1 Raw images — (PDF) [file pone.0263025.s001.pdf]
